# The 2025 Brazilian methanol poisoning crisis: a critical care and emergency medicine review

**DOI:** 10.1590/1806-9282.20251885

**Published:** 2026-05-11

**Authors:** Flavia Gabe Beltrami, Lucas Oliveira Junqueira e Silva, Felippe Leopoldo Dexheimer

**Affiliations:** 1 Hospital de Clínicas de Porto Alegre – Porto Alegre (RS), Brazil.; 2 Hospital Moinhos de Vento – Porto Alegre (RS), Brazil.; 3 Universidade Federal do Rio Grande do Sul, Faculty of Medicine, Department of Internal Medicine – Porto Alegre (RS), Brazil.

## INTRODUCTION

October 2025, emergency departments across Brazil were confronted with a rapidly escalating public health crisis. A surge of patients presenting with unexplained metabolic acidosis, visual disturbances, and profound central nervous system (CNS) depression signaled a massive outbreak of methanol poisoning from adulterated alcoholic beverages. As of October 8, 2025, the Brazilian Ministry of Health had confirmed 24 cases and 5 deaths, with an additional 235 notifications and 11 deaths under investigation nationwide, prompting the declaration of a national public health emergency^1^.

The Brazilian government’s response was swift, involving the immediate distribution of over a thousand units of the antidote ethanol from the national strategic stock, the emergency procurement of 2,600 vials of fomepizole, and the expansion of national diagnostic capacity to 190 analyses per day through reference laboratories like Unicamp and Fiocruz^1^. The Ministry of Health issued a critical directive to clinicians: notify suspected cases immediately and initiate treatment based on clinical suspicion, without awaiting laboratory confirmation^2^.

This crisis, while acute, is not unique. It mirrors other catastrophic mass poisonings, such as the outbreak in Iran during the COVID-19 pandemic, where misinformation led to over 5,800 poisonings and 800 deaths from consuming methanol as a purported prophylactic^3^. These events consistently demonstrate that methanol poisoning is a global challenge for emergency medical systems, disproportionately affecting vulnerable populations through illicitly produced and contaminated spirits.

For the frontline emergency and critical care physician, these outbreaks present a formidable challenge. The stakes are incredibly high, with mortality rates in resource-limited settings exceeding 60%^4^. Survivors often face devastating, life-altering sequelae, including permanent blindness and a debilitating Parkinsonian-like movement disorder from necrotic brain lesions.

This review provides a focused, evidence-based guide for the emergency and critical care management of acute methanol poisoning, drawing lessons from the ongoing Brazilian crisis to underscore the principles of rapid diagnosis, aggressive intervention, and systems-level preparedness.

## PATHOPHYSIOLOGY FOR THE CLINICIAN: FROM METABOLISM TO MULTI-ORGAN FAILURE

Understanding the pathophysiology of methanol poisoning is essential for interpreting its clinical manifestations and guiding therapy. The toxicity is a direct result of its metabolic conversion into formic acid, which triggers a cascade of cellular and systemic insults.

Methanol itself is only a mild CNS depressant. The true danger lies in its hepatic metabolism. The enzyme alcohol dehydrogenase (ADH) converts methanol to formaldehyde, which is then rapidly oxidized by aldehyde dehydrogenase (ALDH) to formic acid^5^. It is the systemic accumulation of this metabolite, formate, that drives the clinical toxidrome. Formate is a potent mitochondrial poison. It directly inhibits cytochrome c oxidase (Complex IV), the final enzyme in the electron transport chain, effectively shutting down aerobic respiration^5^. This leads to two critical consequences for the critically ill patient:

1. Profound cellular hypoxia: Tissues are starved of adenosine triphosphate, leading to cellular dysfunction and death.
2. Severe lactic acidosis: Cells are forced into anaerobic glycolysis, producing lactate that exacerbates the already severe high anion-gap metabolic acidosis caused by formate itself.

While mitochondrial poisoning explains the systemic acidosis, it does not fully account for the selective vulnerability of the retina, optic nerve, and putamen. The modern understanding of methanol neurotoxicity involves a “second hit” mechanism, where the initial metabolic insult triggers a secondary wave of oxidative stress and neuroinflammation. The disruption of the mitochondrial chain leads to a massive overproduction of reactive oxygen species, overwhelming cellular antioxidant defenses^6^. In the CNS, this oxidative damage activates resident immune cells like microglia, which release a flood of pro-inflammatory cytokines. This creates a vicious cycle of inflammation and oxidative injury that culminates in the apoptosis of retinal ganglion cells and neurons within the basal ganglia, leading to the characteristic optic neuropathy and putaminal necrosis. This dual-mechanism pathophysiology underscores why treatment must not only block formate production but also consider strategies to mitigate this secondary neurotoxic cascade.

## CLINICAL RECOGNITION IN THE EMERGENCY DEPARTMENT

The diagnosis of methanol poisoning is frequently missed or delayed due to its nonspecific early symptoms. A high index of suspicion is the most critical diagnostic tool for the emergency physician.

Following ingestion, a latent period of 12–24 h is typical, during which the patient may only report vague symptoms of mild inebriation, headache, nausea, vomiting, or abdominal pain^5^. This period corresponds to the time required to metabolize methanol into toxic levels of formate. A key clinical pearl is that co-ingestion of ethanol competitively inhibits , which can prolong this latent period for up to 96 h, further masking the diagnosis^7^.

As formate accumulates, the classic clinical triad emerges:

1. Visual disturbances: This is the hallmark symptom. Patients report blurred vision, photophobia, or the pathognomonic “snowstorm” vision. Fundoscopy may reveal optic disc hyperemia or papilledema, and pupils may be dilated and nonreactive, a sign of severe optic nerve injury.
2. Altered mental status: Confusion and lethargy can rapidly progress to seizures, coma, and brain death.
3. Metabolic acidosis: As the acidosis worsens, patients develop compensatory hyperventilation, known as Kussmaul respirations.

The emergency physician must recognize signs of impending collapse. The strongest predictors of mortality are a profoundly low arterial pH (<7.0–7.1), a Glasgow Coma Scale score <8, and failure of respiratory compensation (i.e., a rise in the face of severe acidosis), which often precedes terminal respiratory arrest^8^.

## EMERGENCY DIAGNOSTIC STRATEGY: TREATING ON SUSPICION

In a potential methanol poisoning, treatment must be initiated empirically based on clinical suspicion^2,9^. Delaying therapy for confirmatory testing is a critical error that increases morbidity and mortality^7^. This challenge is particularly acute in the context of the Brazilian outbreak, as most healthcare facilities in the country lack the capability to directly measure serum methanol levels, forcing an almost complete reliance on clinical and indirect laboratory clues.

## THE UTILITY OF THE OSMOLAL AND ANION GAPS

In the emergency department (ED), the osmolal and anion gaps are indispensable surrogate markers.

- Osmolal gap (OG):. An elevated OG (>10–25 mOsm/kg) is present early after ingestion when parent methanol levels are high. As methanol is metabolized, the OG falls^7^.
- Anion gap (AG):. A high AG (>12–18 mEq/L) develops later as the toxic metabolite, formate, accumulates^7^.

A key diagnostic concept is their inverse relationship: a patient with a high OG and normal AG is early in the course, while a patient with a normal OG and a profoundly high AG is a late presentation. The presence of both indicates ongoing, active metabolism and requires immediate, aggressive intervention.

## ESSENTIAL LABORATORY AND IMAGING PANEL

The following Table 1 summarizes the essential diagnostic workup for a suspected methanol poisoning.

**Table 1:** Essential diagnostic workup for a suspected methanol poisoning.

Diagnostic tool	Finding in methanol poisoning	Clinical pearls for the ED physician
Arterial blood gas (ABG)	Severe high anion-gap metabolic acidosis with respiratory compensation.	A rising PaCO_2_ is a pre-terminal sign indicating failed compensation and impending respiratory arrest.
Osmolal gap (OG)	Elevated >10–25 mOsm/kg H_2_O	Most useful early. A normal OG in a late-presenting, acidotic patient does not rule out methanol poisoning.
Anion gap (AG)	Elevated >12–18 mEq/L	Rises as formate accumulates. The magnitude of the AG correlates with the severity of poisoning.
Serum ethanol level	May be elevated with co-ingestion.	An elevated ethanol level explains a prolonged latent period and contributes to the OG.
Serum methanol level	>20–25 mg/dL is an indication for treatment.	Do not wait for this result to start treatment. Levels may be low or undetectable in late presentations.

ED: emergency department.

## CRITICAL CARE MANAGEMENT: THE FOUR PILLARS OF THERAPY

Before initiating definitive therapy, it is critical to recognize that traditional gastrointestinal decontamination methods are generally futile. Methanol is a simple alcohol that is absorbed with extreme rapidity from the gastrointestinal tract, with peak serum concentrations occurring within 30–60 min of ingestion^10^. Consequently, gastric lavage or the administration of activated charcoal offers no benefit unless performed almost immediately after ingestion, a scenario rarely encountered in clinical practice. Activated charcoal, in particular, poorly adsorbs alcohols, rendering it ineffective^11^. Therefore, management should bypass these measures and focus directly on the four pillars of systemic therapy.

### Pillar 1: Resuscitation and acidosis correction

Standard ABCs are the priority. Intubate early for airway protection in patients with altered mental status or signs of respiratory fatigue. Aggressive correction of metabolic acidosis with intravenous sodium bicarbonate is crucial for any patient with a pH<7.30. This is not just supportive; it is therapeutic. Alkalinization promotes the conversion of formic acid to its ionized form, formate, which is “trapped” in the plasma and cannot readily cross the blood-brain barrier, thus mitigating neurotoxicity. Additionally, administer cofactor therapy with folinic or folic acid to enhance the endogenous metabolism of formate^10^.

### Pillar 2: Alcohol dehydrogenase inhibition: fomepizole versus ethanol

The definitive medical intervention is blocking ADH to halt formate production^12^.

- Fomepizole: This potent ADH inhibitor is the preferred antidote where available. It has predictable kinetics, a simple dosing regimen, and a vastly superior safety profile.
- Ethanol: This competitive substrate for ADH is an effective alternative but is far more difficult and dangerous to use. It requires a continuous infusion to maintain a therapeutic target (100–150 mg/dL), necessitating frequent monitoring in an intensive care unit (ICU) setting.

One cohort study found adverse drug events (ADEs) in 57% of ethanol-treated patients versus only 12% in the fomepizole group, with common ethanol-related ADEs including CNS depression, hypoglycemia, and hypotension. Furthermore, the complexity of ethanol infusions leads to medication errors in up to 78% of cases^13^.

Despite these drawbacks, a landmark study from a mass poisoning outbreak found no significant difference in clinical effectiveness (mortality or sequelae) between fomepizole and meticulously managed ethanol^14^. This is a critical finding for resource-limited settings like Brazil during the current surge, confirming that ethanol remains a life-saving first-line therapy when fomepizole is unavailable. Table 2 summarizes the characteristics of the enzyme ADH inhibitor agents.

**Table 2:** Characteristics of enzyme alcohol dehydrogenase inhibitor agents.

Feature	Fomepizole	Ethanol
Mechanism	Potent ADH inhibitor	Competitive ADH substrate
Dosing	Simple IV loading and maintenance doses	Complex continuous IV infusion
Monitoring	Clinical monitoring	Frequent serum ethanol levels are required
Adverse events	Low rate (~12%)	High rate (~57%)
Medication errors	Lower risk	High risk (~78%)
Cost	Very high	Low
Clinical efficacy	Equivalent to ethanol when ethanol is properly managed	

ADH: alcohol dehydrogenase.

### Pillar 3: Extracorporeal elimination: intermittent hemodialysis versus continuous renal replacement therapy

Extracorporeal therapy is the definitive treatment for severe poisoning, indicated for severe acidosis (pH<7.25–7.30), visual changes, end-organ damage, or a methanol level >50 mg/dL^10,14^. Its purpose is to rapidly remove both methanol and formate.

- Intermittent hemodialysis (IHD): From a kinetic standpoint, IHD is far superior. Due to higher flow rates, it removes methanol and formate much faster than continuous therapies. The elimination half-life for formate is 1.6 h with IHD versus 3.6 h with CRRT^10,14,15^.
- Continuous renal replacement therapy (CRRT): While kinetically slower, CRRT is a better option for hemodynamically unstable patients.

Crucially, a large prospective study found that after adjusting for admission severity, there was no significant difference in mortality or neurological sequelae between patients treated with IHD versus CRRT^15^. The most critically ill patients are often placed on CRRT, confounding direct comparisons. The vital take-home message for the clinician in a mass casualty setting is “use what you have.” The priority is to initiate any form of extracorporeal therapy as quickly as possible, rather than delaying care to access a specific modality.

The successful implementation of these therapeutic pillars requires a seamless continuum of care that begins in the ED and extends into the ICU. Initial resuscitation, diagnosis, and the initiation of antidotal therapy are the domain of the ED. However, patients with severe poisoning, particularly those requiring continuous ethanol infusions, mechanical ventilation, or extracorporeal therapy, necessitate prompt transfer to an ICU. This transition is a critical juncture where close monitoring and specialized care, including the complex management of hemodialysis, are essential to prevent further deterioration and improve outcomes.

## MASS CASUALTY MANAGEMENT: LESSONS FROM THE BRAZILIAN OUTBREAK

A methanol poisoning outbreak is a mass casualty incident that requires a systems-level public health response. The Brazilian response, involving rapid deployment of antidotes and expansion of lab capacity, provides a model for coordination.

## ACTIVE CASE FINDING AND PUBLIC COMMUNICATION

In an outbreak, the strategy must shift from passive to active case finding. Public warnings via mass media, as used successfully in Iran, can bring asymptomatic but exposed individuals to care early, preventing severe outcomes.

## RESOURCE-STRATIFIED TRIAGE

When resources are overwhelmed, a formal triage system based on principles of distributive justice is essential. A resource-stratified approach should prioritize patients most likely to benefit:

1. Priority 1 (Immediate Dialysis and Antidote): Patients with established poisoning (visual changes, acidosis) but who are not yet moribund.
2. Priority 2 (Antidote and Observation): Asymptomatic patients with a known ingestion or mildly symptomatic patients with normal acid-base status.
3. Priority 3 (Palliative Care): Patients presenting in extremis (e.g., refractory shock, anuria, fixed and dilated pupils). In a resource-scarce scenario, allocating dialysis to these patients may divert it from others with a higher chance of survival.

Consensus guidelines also suggest that it is often more effective to transport mobile dialysis units and expert personnel to an outbreak site rather than attempting to transfer large numbers of unstable patients out.

## CONCLUSION

The 2025 methanol poisoning outbreak in Brazil is a stark reminder that this preventable toxidrome remains a clear and present danger. For the emergency and critical care physician, effective management hinges on a few core principles:

- Maintain a high index of suspicion and initiate treatment empirically without waiting for confirmatory tests.
- Master the interpretation of the osmolal and anion gaps to guide initial therapy.
- Aggressively correct acidosis with sodium bicarbonate.
- Understand the risk-benefit profiles of both fomepizole and ethanol to provide effective ADH blockade regardless of resource availability.
- Initiate extracorporeal therapy urgently for all severely poisoned patients, using whichever modality is available first.

Beyond the individual patient, this crisis calls for the development and implementation of hospital-level mass casualty and triage protocols. Further research is urgently needed to validate adjunctive neuroprotective therapies and to develop rapid, point-of-care diagnostics that could revolutionize management in resource-limited settings^9^. By combining clinical vigilance with robust public health preparedness, the medical community can better combat these recurrent and preventable tragedies.

## Data Availability

The datasets generated and/or analyzed during the current study are available from the corresponding author upon reasonable request.
